# Omitting axillary lymph node dissection in breast cancer patients with extensive nodal disease and excellent response to primary systemic therapy using the MARI protocol

**DOI:** 10.1016/j.breast.2025.104411

**Published:** 2025-02-04

**Authors:** Annemiek K.E. van Hemert, Ariane A. van Loevezijn, Marie-Sophie P.D. Baas, Marcel P.M. Stokkel, Emma J. Groen, Vincent van der Noort, Claudette E. Loo, Gabe S. Sonke, Nicola Russell, Frederieke H. van Duijnhoven, Marie-Jeanne T.F.D. Vrancken Peeters

**Affiliations:** a Department of Surgical Oncology, Netherlands Cancer Institute–Antoni van Leeuwenhoek, Amsterdam, the Netherlands; b Department of Nuclear Medicine, Netherlands Cancer Institute–Antoni van Leeuwenhoek, Amsterdam, the Netherlands; c Department of Pathology, Netherlands Cancer Institute–Antoni van Leeuwenhoek, Amsterdam, the Netherlands; d Department of Biometrics, Netherlands Cancer Institute–Antoni van Leeuwenhoek, Amsterdam, the Netherlands; e Department of Radiology, Netherlands Cancer Institute–Antoni van Leeuwenhoek, Amsterdam, the Netherlands; f Department of Medical Oncology, Netherlands Cancer Institute–Antoni van Leeuwenhoek, Amsterdam, the Netherlands; g Department of Radiation Oncology, Netherlands Cancer Institute–Antoni van Leeuwenhoek, Amsterdam, the Netherlands; h Department of Surgery, Amsterdam UMC–University of Amsterdam, Amsterdam, the Netherlands

**Keywords:** Breast cancer, Primary systemic therapy, Axillary lymph node dissection, Radiotherapy, Recurrence, Survival

## Abstract

**Purpose:**

Axillary lymph node staging techniques after primary systemic therapy (PST) show low false negative rates, stimulating the omission of axillary lymph node dissection (ALND). Breast cancer patients with extensive nodal disease are underreported in studies on response-guided axillary treatment. In this study, we present the oncologic outcome of breast cancer patients with ≥4 involved axillary lymph nodes treated according to the MARI-protocol.

**Methods:**

This prospective single arm registration study included breast cancer patients with extensive nodal disease defined as ≥4 involved axillary lymph nodes on FDG-PET/CT pre-PST between July 2014 and December 2021. After PST, the marked (MARI) lymph node was excised. Patients with a pathologic complete response (pCR) of the MARI node (ypN0) received locoregional radiation treatment (RT). In patients with residual disease of the MARI node (ypN+), ALND was performed followed by RT. Primary endpoint was axillary recurrence rate. Secondary endpoints were invasive disease-free survival (DFS) and overall survival (OS).

**Results:**

In total, 218 patients were registered of which 39 % of patients also had extra-axillary nodal disease. Median (IQR) age was 50 (42–57) years. After PST 47 % of patients (103/218) had ypN0, whereas 53 % of patients (115/218) had ypN+. After a median follow up of 44 (26–62) months, axillary recurrence rate was 2.9 % (*n* = 3) in the ypN0 group and 3.5 % (*n* = 4) in the ypN + group. Five-year invasive DFS and OS were respectively 89 % (95 % CI 83 %–96 %) and 95 % (95 % CI 91 %–99 %) in ypN0 patients.

**Conclusion:**

Omission of ALND after PST in breast cancer patients with extensive nodal involvement who achieve pCR of the MARI node is associated with excellent five-year oncologic outcome.

## Highlights

• In the MARI protocol, patients extensive nodal disease with pCR of the MARI node (ypN0) receive RT only.
• Omission of ALND after PST using the MARI protocol shows excellent oncologic outcome.
• Axillary recurrence rates were low: 2.9 % with RT (ypN0), 3.5 % with ALND + RT (ypN+).
• YpN0 patients treated with RT alone had the highest five year iDFS (89 %) and OS (95 %).

## 1 Introduction

The benefit of axillary lymph node dissection (ALND) in breast cancer patients with extensive axillary lymph node disease treated with primary systemic therapy (PST) remains unclear. ALND is associated with significant morbidity [1,2] and although ALND reduces the rate of locoregional recurrences, it has never been demonstrated to have a positive impact on survival in breast cancer. Tailoring systemic treatment to tumor subtype and biology has led to higher rates of pathologic complete response (pCR) in the breast and axillary lymph nodes. Currently, one third of clinically lymph node positive (cN+) patients achieve axillary pCR after PST [[3], [4], [5], [6], [7], [8]] with highest rates of up to 80 % in patients with hormone receptor (HR) negative/Human Epidermal growth factor Receptor 2 positive (HER2+) breast cancer [6,9]. As axillary pCR is associated with improved oncologic outcome [[10], [11], [12], [13]], opportunities to de-escalate locoregional treatment after PST in cN + breast cancer patients have been extensively explored. Axillary lymph node staging techniques (eg. SLNB = sentinel lymph node biopsy, MARI = Marking one of the pathologic axillary lymph nodes with a Radioactive Iodine seed, and TAD = targeted axillary dissection) after primary systemic therapy (PST) are associated with low false negative rates (FNR) [[14], [15], [16], [17], [18], [19]]. Data on long term oncologic outcome of tailored axillary treatment after axillary re-staging with SLNB or TAD after PST show low axillary recurrence rates ranging from 0 % to 2.8 % with up to ten-year follow up after SLNB [20]. Ongoing randomized controlled trials (Alliance A011202 [21] and OPBC-03/TAXIS [22] and ADARNAT trial [23]) compare ALND and/or locoregional radiotherapy in cN1 patients with residual axillary disease (as assessed with SLNB or TAD). Furthermore, registry studies such as the AXSANA and MINIMAX will contribute to determining the optimal locoregional approach for patients with cN + breast cancer [24,25]. Although patients with more extensive axillary disease achieve similar axillary pCR rates to those with cN1 breast cancer [26], de-escalation of axillary surgery is less investigated in this patient group. All the above-mentioned studies, with promising oncological outcome data, primarily include patients with limited nodal disease (cN1) and patients with extensive nodal disease are underrepresented. The MARI procedure, in which one of the proven axillary lymph nodes is marked with an iodine seed prior to PST and selectively removed after PST, can reliably predict the axillary response with an identification rate of 97 % and FNR of 7 % [27] in patients with limited and in patients with extensive nodal disease. Combining the MARI-procedure with a pre-PST FDG-PET/CT to determine the presence of less or more than three suspicious axillary lymph nodes enabled tailoring the axillary treatment to the response to PST [28,29]. In this study, we present the axillary recurrence rate, estimated five-year invasive disease-free and overall survival for breast cancer patients with extensive nodal disease treated according to the MARI protocol involving omission of ALND in patients with a pCR of the MARI node.

## 2 Methods

### 2.1 Design/participants

This prospective single arm registration study included patients between July 2014 and December 2021 with approval of the Institutional Review Board. All women with pathologically proven cN + breast cancer with ≥4 involved axillary lymph nodes who were treated according to the MARI protocol (= PST followed by tailored axillary treatment according to response of the MARI node) at the Netherlands Cancer Institute – Antoni van Leeuwenhoek (NKI-AVL) were eligible for inclusion.

### 2.2 Diagnostics

All patients underwent core needle biopsies of the breast tumor to determine histological subtype, Bloom-Richardson grade, hormone receptor (HR) status and HER2-expression. The diagnostic work up comprised mammography, ultrasound and dynamic contrast-enhanced (DCE) MRI to determine size and extent of the primary tumor at baseline. Clinical (axillary) nodal disease was confirmed with ultrasound guided fine needle aspiration (FNA) or core needle biopsy. Regional staging and detection of distant metastases was performed with a whole body FDG-PET/CT. Axillary nodal stage was based on the number of FDG-positive axillary lymph nodes (ALNs). Extra-axillary lymph nodes (parasternal/periclavicular) were described separately.

The largest pathologically proven ALN (i.e. MARI node) was marked with an Iodine seed (STM1251, Bard Brachytherapy Inc., Carol Stream, IL) with ultrasound guidance before administration of PST. Adequate position of the seed was confirmed with ultrasound and/or mammography. The MARI procedure and safety protocols were comprehensively described earlier [27].

### 2.3 Treatment and response evaluation

All enrolled patients received PST. Generally, patients with HER2+ disease were treated with nine cycles of paclitaxel, carboplatin, trastuzumab and pertuzumab (PTC-Ptz) or three cycles of 5-fluorouracil, epirubicin, cyclophosphamide, trastuzumab and pertuzumab (FEC-T-Ptz) followed by six cycles of PTC-Ptz. Patients with HER2+ disease treated within the TRAIN-3 trial, received three, six or nine cycles PTC-Ptz depending on radiologic response (NCT03820063). Patients with HR+/HER2-breast cancer received four cycles of bi-weekly (dose-dense) doxorubicin and cyclophosphamide followed by 12 weekly paclitaxel. Patients with TN breast cancer received additional carboplatin concurrent with paclitaxel.

Following PST, surgery of the breast and selective removal of the MARI node was performed. A gamma probe was used to localize the iodine seed for surgical resection. In all patients, intra-operative frozen section of the MARI node was performed; 2 mm tissue slices were made from which 5 μm hematoxylin and eosin (H&E) sections were prepared and assessed. ALND was performed when frozen section showed residual axillary disease. pCR of the axilla is defined as the absence of vital tumor cells in the removed axillary lymph node(s) (ypN0). pCR of the breast is defined as the absence of invasive and in situ carcinoma in the surgical specimen of the breast (ypT0).

### 2.4 Axillary treatment

ALND was omitted in patients with a pCR of the MARI node (ypN0); they were only treated with adjuvant radiation treatment of axillary and periclavicular nodes.

In patients with residual tumour in the MARI node (ypN+) both ALND and locoregional radiation treatment was applied. The target volumes included the breast or chest wall and all axillary levels, the interpectoral nodes and the supraclavicular fossa avoiding the ALND region. Exceptions were patients with ≥10 axillary metastases, extra-nodal extension or nodal ratio of >50 %, when all levels were included in the target volume. In case of a false negative intraoperative frozen section of the MARI node, ALND was performed in a second surgery.

Lymph node levels were delineated following the Danish national delineation guidelines, and from January 2015, adhering to the European Society for Radiotherapy and Oncology consensus guidelines [30]. Prescribed doses were 42.56Gy (16 fractions of 2.66 Gy) or 46.2 Gy (21 fractions of 2.2 Gy) if a simultaneous boost dose was administered to the breast tumor bed. Static field Intensity Modulated RadioTherapy (IMRT) or Volumetic Modulated Art Therapy (VMAT) planning was used to irradiate and Deep Inspiration Breath Hold Technique was used for all left-sided breast tumors to spare the heart.

### 2.5 Adjuvant systemic therapy

Adjuvant systemic therapy was administered according to institutional guidelines. Hormonal therapy was given to all patients with HR + breast cancer and patients with HER2+ disease received a total of 12 months trastuzumab. Adjuvant trastuzumab emtansine (T-DM1) was administered to patients with HER2+ breast cancer with residual disease after PST [31]. Patients with TN breast cancer and residual disease after PST were treated with adjuvant capecitabine [32].

### 2.6 Outcomes

The primary endpoint was the axillary recurrence rate, defined as the percentage of patients with tumor recurrence in ipsilateral axillary lymph nodes. Secondary endpoints were the rate of local, regional and distant metastases, invasive disease-free survival (DFS) and overall survival (OS). Events to calculate invasive DFS include death caused by breast cancer and any other cause, an axillary, local and/or regional recurrence (invasive/DCIS) ipsilateral and contralateral as well as distant metastases [33]. Survival estimates were calculated using the Kaplan-Meier method. All survival estimates were reported with their 95 % confidence intervals. Differences in baseline characteristics between response groups were tested using the Kruskal-Wallis test or Pearson's Chi –square test. A *p*-value <0.05 was considered significant. All statistical analyses were performed in IBM SPSS Statistics, version 25.0.

## 3 Results

### 3.1 Baseline characteristics

Between July 2014 and December 2021, 218 breast cancer patients with ≥4 involved axillary lymph nodes were prospectively registered. Table 1a, Table 1ba,b shows the baseline characteristics by response of the MARI node (ypN0 versus ypN+). Median age was 50 years (IQR 42–57). Median number of involved axillary lymph nodes on pre-PST FDG-PET/CT was five (IQR 4–8) in both patient groups (*p*-value 0.283). 40 % of patients had more than six involved axillary nodes, and pre-PST FDG-PET CT scan showed extra-axillary lymph nodes in 39 % (*n* = 85) of all patients. Supra-clavicular lymph nodes were observed in 6.8 % (*n* = 7) of ypN0 patients and in 15.7 % (*n* = 18) of patients with ypN+ (*p*-value 0.040).

**Table 1a:** Baseline characteristic by response group.

	ypN0		ypN+		Total		*p-value*
Patients	103		115		218		
Age	48	(39–56)	51	(43–58)	50	(42–57)	0.042
Tumor size index lesion (mm)	30	(23–54)	32	(23–53)	31	(23–53)	0.724
cT stage							
*0*	–		1	(0.9 %)	1	(0.5 %)	0.202
*1*	19	(18.4 %)	10	(8.7 %)	29	(13.3 %)	
*2*	50	(48.5 %)	60	(52.2 %)	110	(50.5 %)	
*3*	29	(28.2 %)	41	(35.7 %)	70	(32.1 %)	
*4*	4	(3.9 %)	3	(2.6 %)	7	(3.2 %)	
*is*	1	(1.0 %)	–		1	(0.5 %)	
Involved axillary lymph nodes PET-CT^a^	5	[[4], [5], [6], [7]]	5	[[4], [5], [6], [7], [8]]	5	[[4], [5], [6], [7], [8]]	0.283
Involved axillary lymph nodes PET-CT, categorised^a^							0.558
*4*	33	(38.8 %)	30	(33.3 %)	63	(36.0 %)	
*5*	21	(24.7 %)	20	(22.2 %)	41	(23.4 %)	
*≥6*	31	(36.5 %)	40	(44.4 %)	71	(40.6 %)	
Extra axillary lymph nodes							0.815
*None*	62	(60.2 %)	71	(61.7 %)	133	(61.0 %)	
*Infraclavicular*	18	(17.5 %)	13	(11.3 %)	31	(14.2 %)	
*Supraclavicula*	7	(6.8 %)	18	(15.7 %)	25	(11.5 %)	
*Parasternal*	16	(15.5 %)	13	(11.3 %)	29	(13.3 %)	
Histology							<0.001
*NST*	101	(98.0 %)	96	(83.5 %)	197	(90.4 %)	
*ILC*	1	(1.0 %)	19	(16.5 %)	20	(9.2 %)	
O*ther*	1	(1.0 %)	0	(0.0 %)	1	(0.4 %)	
Subtype							<0.001
*HR+/HER2-*	12	(11.7 %)	74	(64.3 %)	86	(39.4 %)	
*HR+/HER2+*	29	(28.2 %)	12	(10.4 %)	41	(18.8 %)	
*HR-/HER2+*	34	(33.0 %)	2	(1.7 %)	36	(16.5 %)	
*TN*	28	(27.2 %)	27	(23.5 %)	55	(25.2 %)	
Grade^b^							0.010
*1*	1	(1.0 %)	4	(3.9 %)	5	(2.5 %)	
*2*	35	(36.1 %)	55	(53.4 %)	90	(45.0 %)	
*3*	61	(62.9 %)	44	(42.7 %)	105	(52.5 %)

**Table 1b:** Surgical outcome and pathology results by response group.

	**ypN0**		**ypN+**		**Total**	
Surgery						
*BCS*	71	(68.9 %)	59	(51.3 %)	130	(59.6 %)
*Mastectomy*	32	(31.1 %)	55	(47.8 %)	87	(39.9 %)
ypT^c^						
*0*	77	(75.5 %)	8	(7.0 %)	85	(39.2 %)
*1*	15	(14.7 %)	55	(47.8 %)	70	(32.2 %)
*2*	2	(2.0 %)	24	(20.9 %)	26	(12.0 %)
*3*	2	(2.0 %)	24	(20.9 %)	26	(12.0)
*4*	0	(0.0 %)	0	(0.0 %)	0	(0.0 %)
*is*	6	(5.9 %)	3	(2.6 %)	9	(4.1 %)
ypN						
*0*	103	(100.0 %)	0	(0.0 %)	103	(47.2 %)
*0i+*			4	(3.5 %)	4	(1.8 %)
*1*			36	(31.3 %)	36	(16.5 %)
*2*			53	(46.1 %)	53	(24.3 %)
*3*			22	(19.1 %)	22	(10.1 %)
Number of removed lymph nodes (MARI procedure)	1	[1,2]	1	[1,2]	1	[1,2]
Number of removed lymph nodes (ALND)			14	[[10], [11], [12], [13], [14], [15], [16], [17], [18], [19]]		
Number of positive lymph nodes			5	[[2], [3], [4], [5], [6], [7], [8]]		
Adjuvant therapy						
*Endocrine*	40	(38.8 %)	86	(74.8 %)	126	(57.8 %)
*Chemotherapy*	2	(1.9 %)	58	(50.4 %)	61	(28.0 %)
*Targeted therapy*	63	(61.2 %)	18	(15.7 %)	80	(36.7 %)
Follow-up	45	(28–60)	44	(26–65)	44	(26–62)

Numbers are in *n* (%) and median (IQR). Abbreviations: ypN0, pathologic complete response of the MARI node; ypN+, pathologic complete response of the MARI-node; MARI, Marking Axillary lymph nodes with Radioactive Iodine seeds; ALND, axillary lymph node dissection; HR, hormone receptor; HER2, Human Epidermal growth factor 2. ^a^n unspecified in 18 patients in the ypN0 group, unspecified in 25 patients in the ypN + group. ^b^ 18 missing values. ^c^ 2 missing values.

Histology showed invasive breast cancer of no special type (NST) in the majority (*n* = 197, 90.4 %) of patients. Molecular subtypes differed between groups: in ypN0 11.7 % (*n* = 12) was diagnosed with HR+/HER2-subtype, 28.2 % (*n* = 29) with HR+/HER2+ subtype, 33 % (*n* = 34) with HR-/HER2+ subtype and 27.2 % (*n* = 28) with TN subtype. Most ypN + patients were diagnosed with HR+/HER2-breast cancer (*n* = 74, 64.3 %), 10.4 % (*n* = 12) with HR+/HER2+ subtype, two patients (1.7 %) with HR-/HER2+ subtype and 23.5 % (*n* = 27) with TN subtype (*p*-value <0.001).

### 3.2 Pathology results

A median of one (IQR 1–2) lymph node was removed during the MARI procedure. In 50.9 % (*n* = 111), the MARI node showed a pCR on frozen section. In eight of these patients the frozen section was false negative (7.2 %), thus in total, 103 (47.2 %) patients had ypN0. Most patients (*n* = 77, 74.7 %) with ypN0 achieved pCR of the breast as well. In the remaining patients (*n* = 26, 25.3 %), 20 patients had residual invasive carcinoma in the breast and in six patients only DCIS was detected.

In 115 (52.8 %) ypN + patients who underwent ALND, a median number of 14 (IQR 10–19) lymph nodes were removed during the ALND. In the majority of patients (*n* = 101, 87.8 %) additional positive nodes were found; the median number of additional positive lymph nodes was five (IQR 2–8). In patients with ypN+, only 7 % (*n* = 8) achieved pCR of the breast; 103 patients had residual invasive carcinoma in the breast and three patients had DCIS.

Locoregional treatment and adjuvant systemic treatment.

ALND was omitted in all 103 patients with ypN0 and these patients received RT only. All 115 ypN + patients underwent ALND and RT.

The majority (*n* = 83, 80.6 %) of ypN0 patients received adjuvant systemic treatment: two patients were treated with adjuvant chemotherapy due to residual disease of the breast, 40 (38.8 %) patients were treated with endocrine treatment and 63 (61.2 %) with targeted therapy. Almost all patients with ypN+ were treated with adjuvant systemic therapy (*n* = 109, 94.8 %): chemotherapy was administered to 58 (50.4 %) patients, 86 (74.8 %) patients were treated with endocrine treatment and 18 (15.7 %) patients with targeted therapy.

### 3.3 Recurrence rate

After a median follow up of 44 months (IQR 26–62), locoregional (including axillary) recurrences occurred in six (5.8 %) patients with ypN0 and in 15 (13.0 %) patients with ypN+ (Table 2). The axillary recurrence rate was 2.9 % (*n* = 3) in ypN0 patients after RT alone and 3.5 % (*n* = 4) in ypN + patients treated with ALND and RT. Two out of three axillary recurrences in the ypN0 group were isolated recurrences which were treated with ALND and salvage mastectomy. In the third patient, axillary recurrence occurred simultaneously with a distant metastasis and palliative care was initiated. Distant metastases were detected in six (5.8 %) patients with ypN0 and in 16 (13.9 %) patients with ypN+. Overall recurrence rate was 9.7 % (*n* = 10) in ypN0 patients; recurrences occurred in two patients with HR+/HER2+, four patients with HR-/HER2+ and four patients with TN breast cancer. In patients with ypN + recurrence rate was 20.9 % (*n* = 24); 12 patients with HR+/HER2-breast cancer developed a recurrence as well as three patients with HR+/HER2-and nine with TN breast cancer. Supplementary Table 1 shows the location of recurrences in patients with extra-axillary lymph nodes (*n* = 85) only.

**Table 2:** Location of recurrences.

	ypN0	ypN+	Total
	(n = 103)	*(n* = 115)	
Axillary + Local	2	0	2
Axillary + Regional	0	1	1
Axillary + Distant	1	3	4
Local	1	2	3
Local + Regional	0	1	1
Regional	1	4	5
Regional + Distant	1	4	5
Distant	4	9	13
Total	10	24	34
Axillary	3	4	7
Locoregional	6	15	21
Distant	6	16	22

Abbreviations: ypN0, pathologic complete response of the MARI node; ypN+, pathologic complete response of the MARI-node; MARI, Marking Axillary lymph nodes with Radioactive Iodine seeds; LRRT, locoregional radiotherapy; ALND, axillary lymph node dissection.

### 3.4 Survival

**Fig. 1:**
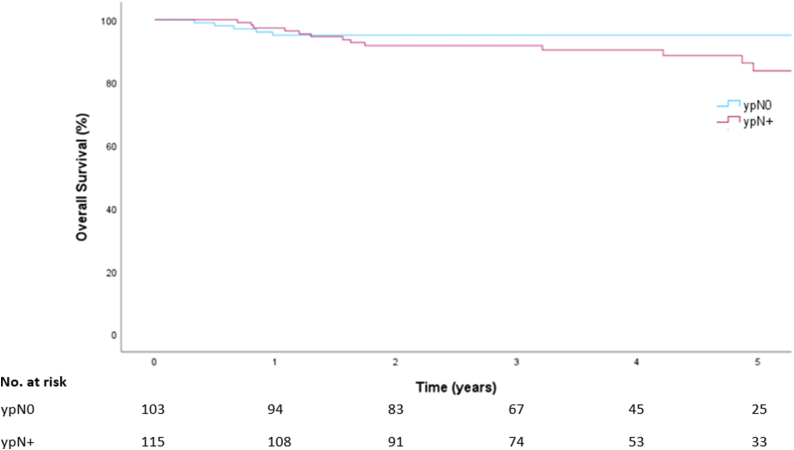
Invasive disease free survival by response group. Abbreviations: ypN0, pathologic complete response of the MARI node; ypN+, pathologic complete response of the MARI-node.

**Fig. 2:**
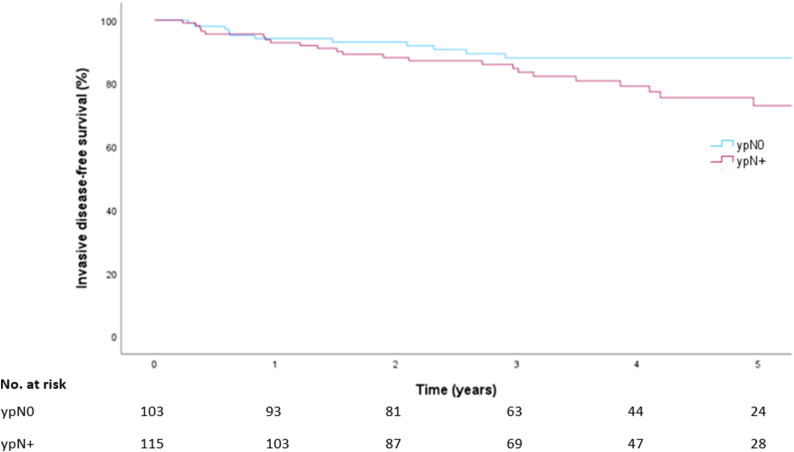
Overall survival by response group. Abbreviations: ypN0, pathologic complete response of the MARI node; ypN+, pathologic complete response of the MARI-node.

Median follow up was 44 months (IQR 26–62). Invasive DFS per response group is shown in Fig. 1. Estimated five-year invasive DFS was 89 % (95 % CI 83 %–96 %) in patients with ypN0 and 76 % (95 % CI 67 %–86 %) in ypN+. Estimated five-year OS per response group is shown in Fig. 2; OS was 95 % (95 % CI 91 %–99 %) in patients with ypN0 and 84 % (95 % CI 75 %–93 %) in patients with ypN+.

## 4 Discussion

This study shows that omission of ALND can be discussed in breast cancer patients with extensive nodal disease with a pCR of the MARI node after PST. We found good oncologic outcome with an axillary recurrence rate of 2.9 % (*n* = 3), a five-year invasive DFS of 89 % (95 % CI 83 %–96 %) and OS of 95 % (95 % CI 91 %–99 %) after a median follow up of 44 months (IQR 26–62).

In the MARI protocol, the number of involved axillary lymph nodes was used for patient classification, as a higher number of involved axillary lymph nodes is a well-established risk factor for locoregional recurrence [[34], [35], [36], [37]]. Moreover, patients with ≥4 involved axillary lymph nodes receive radiotherapy regardless of their response to PST [38]. Given that 39 % (*n* = 85) of patients had extra-axillary nodal disease, this cohort represents a high risk group.

Nowadays, axillary staging techniques are increasingly applied in cN + breast cancer patients. While there is considerable heterogeneity in the surgical approach of the axilla, in the majority (70 %) of cN1 patients with ≤3 involved lymph nodes who convert to ycN0, ALND is not performed anymore [39]. However, among patients with more extensive nodal disease necessitating locoregional radiation treatment, ALND is still standard of care, irrespective of the axillary nodal response [39]. As ALND is associated with significant and frequently persistent morbidities, such as lymphedema and limited shoulder mobility, especially in combination with RT, it should be performed only when beneficial.

Although cN + stage is an important predictor of oncological outcome in breast cancer patients, axillary pCR has a greater impact [40].

A Dutch population-based study of 18.458 invasive breast cancer patients treated with PST with a median follow up of 5.6 years reported significant association of cN + stage as well as ypN + stage with worse OS [41]. They compared five year OS between ypN0 and ypN+ in cN1, cN2 and cN3 subgroups. In all subgroups ypN0 patients had statistically better OS compared to ypN + patients. The cN2 and cN3 subgroup showed a five year OS of respectively 88.9 % (95 % CI 83.7–92.5) and 84.2 % (95 % CI 81.0–87.0) in case of ypN0 and a five year OS of respectively 67.9 % (95 % CI 62.4–72.8) and 65.6 % (95 % CI 62.3–68.7) in case of ypN+ (*p*-value <0.0001) [41].

Alongside the good oncologic outcome we report in the present study (axillary recurrence rate 2.9 % (*n* = 3), OS 95 % (95 % CI 91 %–99 %)), the previous mentioned data support the strategy of de-escalating axillary surgery in patients achieving nodal pCR, also in patients with extensive nodal disease.

Limitations of our study include the use of radioactive iodine seeds in the MARI-procedure. Although iodine seeds provide a safe and effective method for tumor localization [42] and are increasingly being used, their handling and disposal is often subject to strict regulations. In our protocol, iodine seeds are routinely placed before the start of PST and remain in situ for the duration of PST, alternative methods such as clipping the node prior to PST allowing for Iodine seed placement only after PST could overcome some regulatory problems. In this study, FDG-PET/CT was used to stage the axilla pre-PST. As FDG-PET/CT is not yet part of the diagnostic work-up for cN + patients in several countries, the costs may not always be fully covered by health insurances. However, previous studies have shown that FDG-PET/CT is the optimal locoregional staging method with a positive predictive value (PPV) of 98 % [43] for detecting axillary metastases, outperforming US (PPV of 70 %) and MRI (PPV of 76–78 %) [44]. Also, as FDG-PET/CT accurately assesses distant disease, conventional imaging like computed tomography, chest X-ray, bone scans, and liver ultrasound is unnecessary, improving cost-effectiveness [45].

In conclusion, in this study we demonstrated that omission of ALND after PST in patients with ≥4 involved lymph nodes achieving an axillary pCR resulted in an axillary recurrence rate of 2.9 %, five-year invasive DFS of 89 % and an excellent five year OS of 95 %. Therefore, omission of ALND can be discussed in patients with extensive nodal disease achieving axillary pCR.

## CRediT authorship contribution statement

**Annemiek K.E. van Hemert:** Writing – original draft, Formal analysis, Data curation. **Ariane A. van Loevezijn:** Writing – review & editing. **Marie-Sophie P.D. Baas:** Writing – review & editing, Data curation. **Marcel P.M. Stokkel:** Writing – review & editing. **Emma J. Groen:** Writing – review & editing. **Vincent van der Noort:** Writing – review & editing, Formal analysis. **Claudette E. Loo:** Writing – original draft. **Gabe S. Sonke:** Writing – review & editing. **Nicola Russell:** Writing – review & editing. **Frederieke H. van Duijnhoven:** Writing – review & editing, Supervision. **Marie-Jeanne T.F.D. Vrancken Peeters:** Writing – review & editing, Supervision, Methodology, Funding acquisition, Conceptualization.

## Ethical approval

Ethical approval for this study was obtained by the Institutional Review Board of the Netherlands Cancer Institute.

## Funding and conflict of interest

This work was supported by Reggeborgh Foundation, Herja Foundation and AVL Foundation with funding of a PhD student. GS reports institutional research support from Agendia, AstraZeneca, Merck, Novartis, Roche and Saegen, unrelated to this manuscript. Given her role as Editor, MVP had no involvement in the peer review of this article and has no access to information regarding its peer review. All other authors declare no competing interests.
