# Supplementary material for: Omitting axillary lymph node dissection in breast cancer patients with extensive nodal disease and excellent response to primary systemic therapy using the MARI protocol
Source: Breast. 2025 Feb 4;80:104411. doi: 10.1016/j.breast.2025.104411 (PMC11872389; doi:10.1016/j.breast.2025.104411)
Supplement: Multimedia component 1 [file mmc1.docx]

**Supplementary**

| **Table 1. Location of recurrences in patients with extra-axillary disease (*n* = 85)** | | | | | |
| --- | --- | --- | --- | --- | --- |
|  | **ypN0**  **(*n* = 41)** | **ypN+**  ***(n* = 44)** | |  | |
|  |  |  | | **Total** | |
|  |  |  | |  | |
| Axillary + Local | 1 | | 0 | | 1 |
| Axillary + Regional | 0 | | 1 | | 1 |
| Axillary + Distant | 0 | | 2 | | 2 |
| Local | 0 | | 1 | | 1 |
| Local + Regional | 0 | | 1 | | 1 |
| Regional | 1 | | 2 | | 3 |
| Regional + Distant | 1 | | 2 | | 3 |
| Distant | 4 | | 5 | | 9 |
| Total | 7 | | 14 | | 21 |
| Axillary | 1 | | 3 | | 4 |
| Locoregional | 3 | | 9 | | 12 |
| Distant | 5 | | 9 | | 14 |
| Abbreviations: ypN0, pathologic complete response of the MARI node; ypN+, pathologic complete response of the MARI-node; MARI, Marking Axillary lymph nodes with Radioactive Iodine seeds; LRRT, locoregional radiotherapy; ALND, axillary lymph node dissection | | | | | |
